# “Mulligan Bent Leg Raise” Technique in Avascular Necrosis

**DOI:** 10.7759/cureus.50727

**Published:** 2023-12-18

**Authors:** Samruddhi M Karanjkar, Pooja Dhage

**Affiliations:** 1 Physiotherapy, Ravi Nair Physiotherapy College, Datta Meghe Institute of Medical Sciences, Wardha, IND; 2 Musculoskeletal Physiotherapy, Ravi Nair Physiotherapy College, Datta Meghe Institute of Medical Sciences, Wardha, IND

**Keywords:** range of motion, femoral head, physiotherapy, straight leg raise, avascular necrosis, mulligan bent leg raise technique

## Abstract

This case report examines the effectiveness of the “Mulligan bent leg raise” (MBLR) method for treating femoral head avascular necrosis (AVN). A professional physiotherapist directed a six-week rehabilitation program for a 37-year-old male patient with AVN that included this innovative physiotherapeutic method. According to radiographic results and standardized evaluations, the patient showed significant improvements in hip range of motion, functional ability, and pain levels. As a viable supplement to conventional rehabilitation techniques, the MBLR method has shown promise in improving joint function and reducing symptoms in individuals with acute compartment syndrome. The present study provides significant contributions to the discipline. It highlights the necessity for more investigation to examine the wider relevance and enduring effectiveness of this methodology in various population affected.

## Introduction

Avascular necrosis (AVN) is a cytotoxic necrosis or ischemia of the epiphyseal bone that often affects young people, causing pain in the joints and reducing average lifetime. AVN highlights the possibility of a systemic etiology and can be either unifocal or multifocal [1]. The femoral head is affected by AVN more than 75% of the time [1]. AVN of the femoral head (AVNFH) develops when the blood supply of the proximal femur is interrupted. A traumatizing incident may have caused an impact on blood flow, or they may have a non-traumatic reason [2]. AVNFH is a prevalent condition that has detrimental implications for people and healthcare organizations [3] and is marked by the death of osteocytes (bone marrow, bone-forming, and bone-destroying cells), which causes the bone to break up. The upper mantle cartilage is then affected, obliterating the head’s outer layer and ultimately leading to secondary osteoarthritis [4]. AVNFH is a severe condition marked by the loss of bone fibers, which impairs the healing of compressive stress in the femoral head. This may eventually cause the structural bony component of the femoral head to disintegrate, resulting in joint discomfort and performance deterioration [5]. AVNFH progresses through distinct stages. In stage I, there are no observable findings. Stage II is when cystic and sclerotic alterations start to show. Stage III of the illness is characterized by subchondral flattening and collapse. Lastly, distortion of the acetabulum and femoral head occurs in stage IV. These stages represent the progressive deterioration of the femoral head and provide a framework for understanding the severity and extent of AVNFH [6]. The emergence of AVN has been considered linked to particular risk factors, such as smoking, using corticosteroids, having diabetes, rheumatoid arthritis, systemic lupus erythematosus, and sickle cell disease, a few of those [7]. In the initial phases of the disease, AVNFH is challenging to identify on plain radiographs [8].

Pathophysiology of AVN

Carpal bones and other osseous regions of the skeleton are the most often affected areas of the complex and diverse illness known as AVN. It now affects all carpal bones, though it is mostly found in lunates (Kienbock disease) and scaphoid patients (Preiser disease). AVN can have a wide range of causes, but they often fall into one of three groups: extravascular compression, intravascular obliteration, or direct vascular disruption. There are several earlier studies from which to draw conclusions, notwithstanding the paucity of studies on the pathophysiology of carpal-specific AVN in the literature to date. The vascular architecture of the carpal bones has been the subject of the most persuasive research [9].

Mulligan bent leg raise technique

In individuals with referred thigh pain or AVN, the Mulligan bent leg raise (MBLR) technique is stated as a method of improving straight leg raise (SLR). This method aims to improve physical ailments while restoring the standard range of motion (ROM). The MBLR procedure may have improved the SLR range by mobilizing the painful, sensitive nerve tissues [10]. The MBLR method involved five increasingly more flexed hip postures for three repetitions of pain-free, isometric hamstring contraction [11].

The procedure of the MBLR technique

The therapist was standing at the restricted SLR area of the patient. The therapist maintained the patient’s knee bent over his shoulder. The patient is told to push the therapist away with their leg. The patient’s bent knee was now stretched as far as it could go in the direction of the ipsilateral shoulder by the therapist. The leg was stretched out for a considerable period (between 7 and 10 seconds), after which it was returned to the couch. For two weeks, this method was practiced five days a week, three times every session, every day [12].

## Case presentation

Patient findings 

A 37-year-old male patient, labor by occupation with left-hand dominance, reported to the rehabilitation center with complaints of pain in the left lower limb and limited ROM for one month. He mentioned a history of a fall one year ago at a working site, and he experienced pain in his left hip and was treated with medications. The patient was all right for several weeks before he began to feel pain in his left hip. The pain was gradual at the start and progressive, with a severity of 8 out of 10 on the numerical pain rating scale (NPRS) when moving and 2 out of 10 while resting. He reported dull aching that became worse with exertion and mobility and went away with rest and medication. With time, the pain gradually worsened, making it challenging for the patient to bend or sit cross-legged. The patient complained of discomfort in his left hip and trouble stepping, so he proceeded to the orthopedics medical center. An X-ray was advised by the orthopedic specialist, and AVN in the left femoral head was identified. Because of the left hip’s restricted or limited ROM, physiotherapy was recommended.

Clinical findings

**Figure 1:**
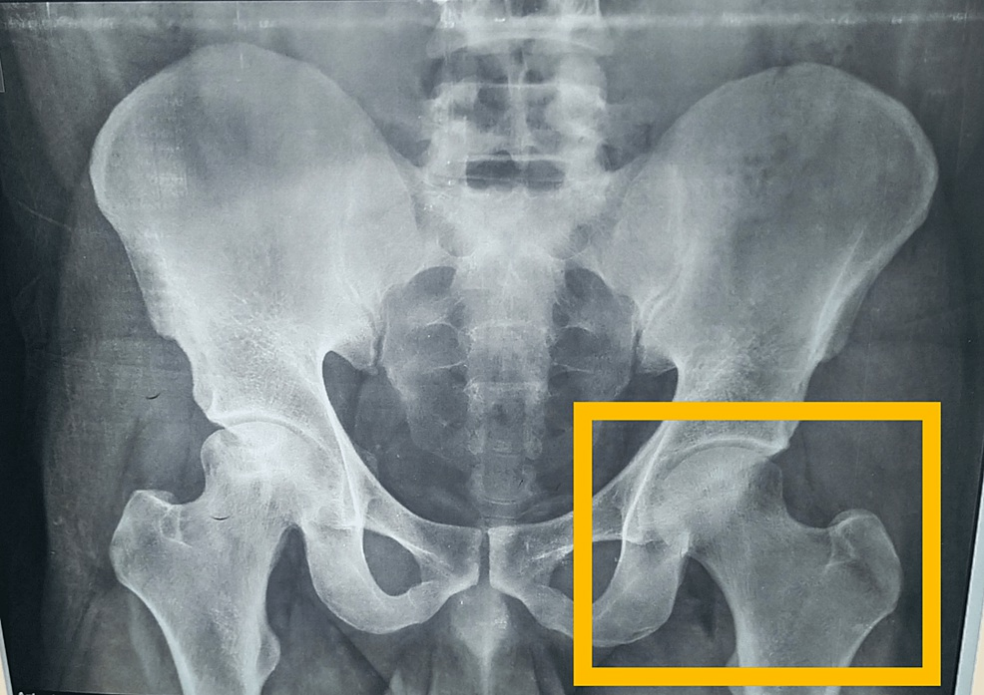
X-ray showing PA view of the hip joint with sclerotic and erosive changes in the left head of the femur

**Figure 2:**
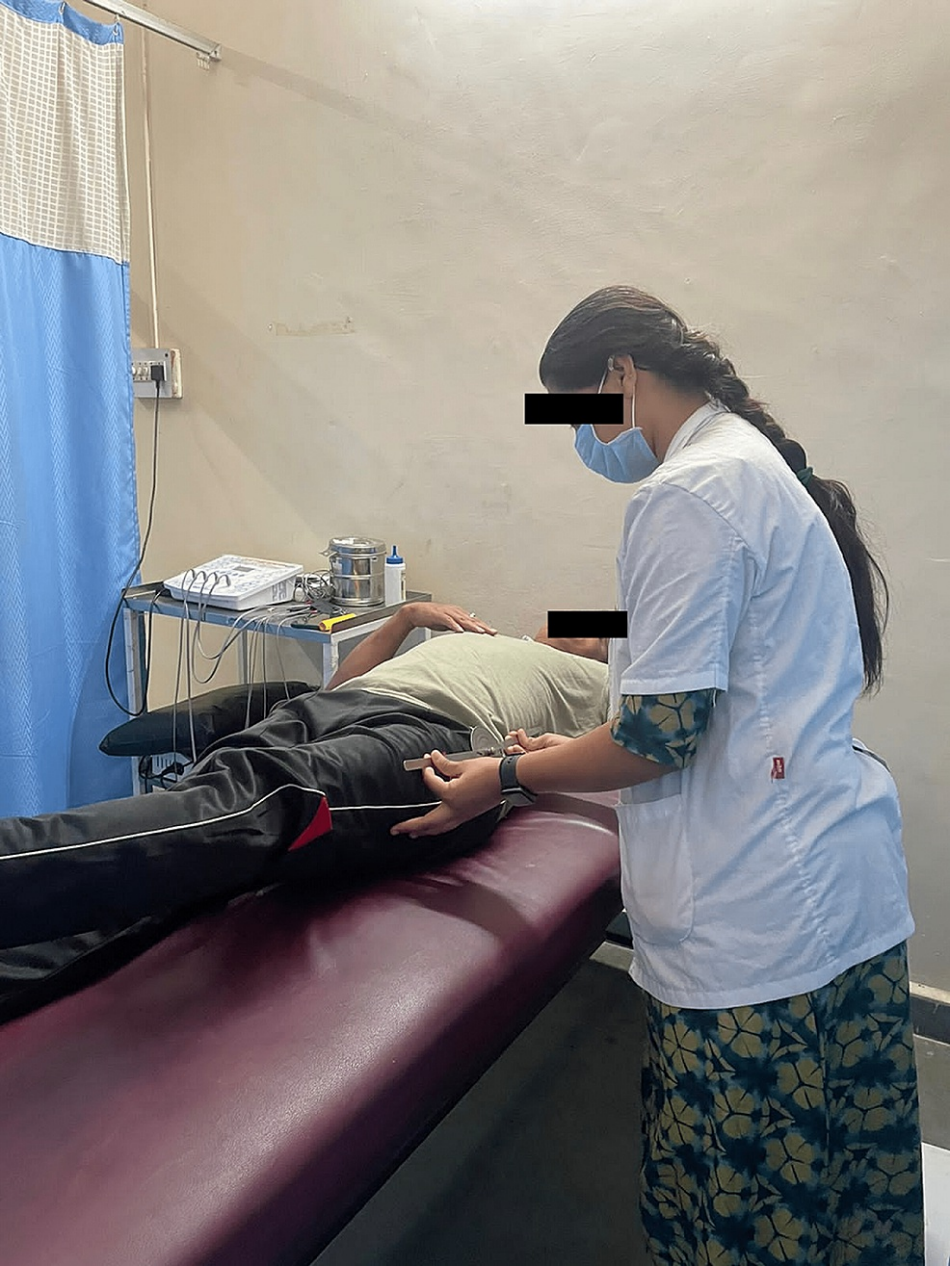
ROM assessment using a goniometer ROM, range of motion

The patient was cooperative, conscious, and well-oriented to time, location, and people. The patient had reduced left hip abduction and hip hiking. Palpation revealed no rise in the local temperature, and grade 2 tenderness was detected just above the left anterior joint line of the hip. Muscle atrophy was also visible in the left thigh. The hamstring and abductor muscles on the affected side were taut, and special tests such as the 90-90 hamstrings and Patrick’s test were also positive. The distal extremity’s circulation was intact. The hamstring and abductor muscles of the affected side were tight. Both sides revealed normal reflexes and sensory assessments throughout the neurological interpretation. Gait analysis revealed a modified gait pattern with a decreased foot flat in the stance phase. Figure 1 shows the patient’s X-ray. ROM is assessed using a goniometer, as shown in Figure 2.

Outcome measures before and after an intervention, such as NPRS, for evaluation of pain are seen in Table 1. Table 2 shows the ROM assessment, and Table 3 shows manual muscle testing (MMT) scores.

**Table 1:** NPRS for pain assessment NPRS, numerical pain rating scale

	Pre-treatment		Post-treatment	
Sr No.	Score on rest	Score on activity	Score on rest	Score on activity
1.	3/10	8/10	2/5	5/10

**Table 2:** ROM of left lower limb ROM, range of motion

		Pre-treatment		Post-treatment	
Sr No.	Movement	Active ROM (degrees)	Passive ROM (degrees)	Active ROM (degrees)	Passive ROM (degrees)
1.	Hip flexion	0-25	0-30	0-110	0-130
2.	Hip extension	0-50	0-55	0-55	0-60
3.	Hip abduction	0-20	0-20	0-45	0-45
4.	Knee flexion	0-120	0-125	0-130	0-135
5.	Knee extension	120-0	125-0	130-0	135-0

**Table 3:** MMT of the left lower limb MMT, manual muscle testing

Sr No.	Muscle group	Score (pre)	Score (post)
1.	Hip flexors	3/5	4/5
2.	Hip extensors	3/5	4/5
3.	Hip abductors	3/5	4/5
4.	Knee flexors	4/5	5/5
5.	Knee extensors	4/5	5/5

Therapeutic intervention

**Figure 3:**
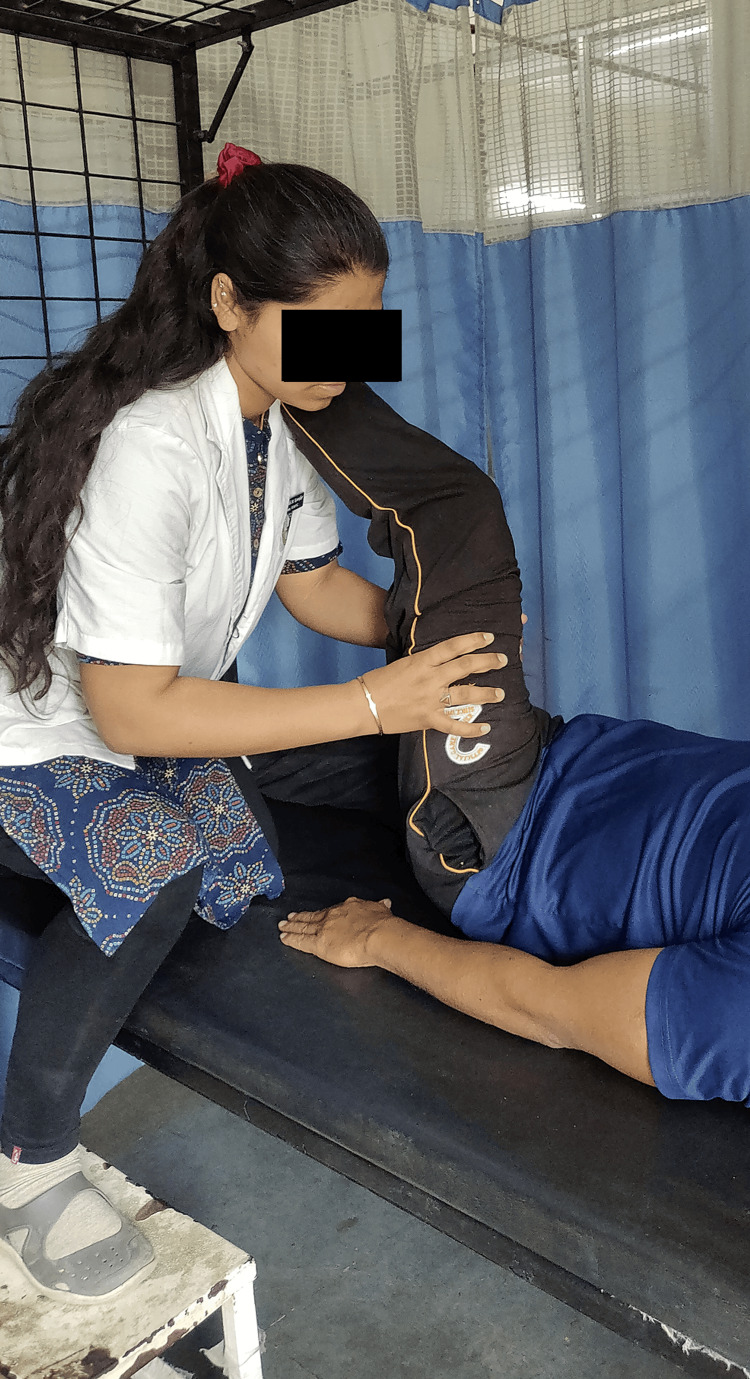
Therapist performing Mulligan bent leg raise technique

Therapeutic intervention included patient education of the condition and the need for physiotherapy treatments to ease symptoms. They decreased both the resting and active pain scores on the NPRS. They helped to improve and maintain ROM for all the affected motions and to ensure that the patient’s lower limb muscle power was enhanced and preserved. They help the patient become self-sufficient and improve their functional capacity. Three repetitions of the MBLR approach were performed. After holding this stretch for 7-10 seconds, it was released [12]. Five treatment sessions each week were part of the two-week strategy. Figure 3 shows the therapist demonstrating the MBLR method.

## Discussion

In the case report, a 37-year-old man has been diagnosed with AVN of the hip joint, a disorder that results in degeneration of the bone and joint due to a weakened blood supply. The patient’s ROM was restricted, and his hip discomfort was getting worse. A two-week rehabilitation program that included the MBLR method under the guidance of a qualified physiotherapist was started in response to these symptoms.

Hall et al., in their study, examined the impact of the MBLR technique on ROM and pain in individuals with limited SLR and low back pain (LBP) [11]. Despite no immediate difference in the SLR range, the BLR group resulted in a notable 7° increase in the range 24 hours post-intervention. However, pain reduction was noted overall, indicating the need for further research to confirm its effectiveness [11]. Adnan et al., in their article, concluded that both the neurodynamic and the bent leg raise method dramatically decreased pain, intellectual deficits, and SLR range in people with LBP that travels downward to the knees [12]. AVN occurs due to an alteration in blood supply to the femoral head [13]. Many physiotherapeutic approaches are used to treat AVN; however, this report enlightens the benefits of using the MLBR technique in addition to other conservative strategies. Improved outcomes are also linked to the Mulligan bent leg lift technique, which is used toward the conclusion of the ROM and keeps the hip joint in its natural position. The Mulligan bent leg lift method can, therefore, be used to increase the hip ROM or reduce stiffness in the hip joint.

Tambekar et al., in their article, concluded that both the Mulligan and Butler approaches demonstrated that SLR and discomfort were reduced right after the intervention [14]. In their paper, Athanasiadis et al. came to the conclusion that there is currently little and conflicting data supporting the biopsychosocial benefits of Mulligan procedures for treating patients with LBP [15]. More research on the bio-psychosocial components of the Mulligan will strengthen the body of data supporting the management of LBP patients with manual therapy and enhance clinical judgment when it comes to non-pharmacological treatment options [15].

LBP is a widespread ailment that affects individuals of all ages. The method of therapy relies on the reason for the pain, which might have a variety of origins. Exercises for overall power have been shown to reduce LBP. Numerous research has been done to examine the effectiveness of different LBP therapeutic approaches, such as the McKenzie technique, Mulligan mobilization, and aquatic therapies. We learned from this review that LBP is a problem that everybody deals with. In comparison to other manual therapy methods, Mulligan mobilization is a more effective course of treatment for persistent LBP. When concluding, factors including pain, stiffness, and impairment were taken into account [16].

## Conclusions

This case report’s evaluation of the MBLR approach in the setting of AVN points to its potential as a creative and useful intervention. The good results, which included a considerable increase in the patient’s hip ROM, functional ability, and pain levels, highlight the potential advantages of implementing this approach into AVN rehabilitation regimens. Even though this case study offers insightful information, it is critical to recognize that more investigation and larger-scale studies are required to confirm the effectiveness and generalizability of the MBLR approach across a range of AVN-affected people. However, these preliminary results motivate physicians and physiotherapists to contemplate this innovative methodology as a beneficial supplement to conventional rehabilitation techniques.
